# 超高效液相色谱-串联质谱法测定灰尘中26种双酚类化合物

**DOI:** 10.3724/SP.J.1123.2022.08022

**Published:** 2023-05-08

**Authors:** Jialin SUN, Yumin NIU, Qun GAO, Jing ZHANG, Bing SHAO

**Affiliations:** 1.首都医科大学公共卫生学院,北京100069; 1. School of Public Health, Capital Medical University, Beijing 100069, China; 2.北京市疾病预防控制中心,食物中毒诊断溯源技术北京市重点实验室,北京100013; 2. Beijing Key Laboratory of Diagnostic and Traceability Technologies for Food Poisoning, Beijing Center for Disease Prevention and Control, Beijing 100013, China; 3.北京市疾病预防控制中心,应急办公室,北京100013; 3. Department of Emergency Management, Beijing Center for Disease Prevention and Control, Beijing 100013, China

**Keywords:** 超高效液相色谱-串联质谱, 双酚类化合物, 灰尘, ultra performance liquid chromatography-tandem mass spectrometry (UPLC-MS/MS), bisphenols (BPs), dust

## Abstract

建立了一种超高效液相色谱-串联质谱(UPLC-MS/MS)同时测定灰尘中26种双酚类化合物(BPs)的多残留分析方法。实验通过优化26种BPs的色谱-质谱参数,比较了不同色谱柱和流动相的分离效果,同时考察了提取溶剂、固相萃取(SPE)等前处理条件对目标化合物的提取效率和净化效果的影响,再结合同位素内标法进行定量,实现了对灰尘中26种BPs的定量分析。灰尘样品依次用3 mL乙腈和3 mL 50%甲醇水溶液进行超声萃取,合并两次提取液,用6 mL超纯水稀释后通过Oasis HLB(60 mg/3 mL)固相萃取小柱,0.5 mL 40%甲醇水溶液淋洗,2 mL甲醇洗脱。以甲醇-1 mmol/L氟化铵水溶液为流动相,流速为0.3 mL/min,经CORTECS^®^ UPLC^®^ C_18_色谱柱(100 mm×2.1 mm, 1.6 μm)分离后,在电喷雾正、负离子多反应监测(MRM)模式下检测26种BPs。26种目标物在各自的范围内线性关系良好,相关系数(*r*^2^)>0.999,方法的检出限(LOD)为0.01~0.75 μg/kg,定量限(LOQ)为0.02~2.50 μg/kg。按LOQ、2倍LOQ和10倍LOQ 3个水平进行加标回收试验,回收率为83.7%~114.9%,日内相对标准偏差(RSD)为0.86%~9.79%(*n*=6),日间RSD为5.16%~19.5%(*n*=6)。对北京市生活区的11份灰尘样品进行检测,检出5种化合物。其中双酚A(BPA)、双酚S(BPS)、双酚F(BPF)、4-羟基-4'-异丙氧基二苯砜(BPSIP)和二苯砜(DPS)5种化合物的检出率为100.0%。该方法精密度好,灵敏度高,可对灰尘中26种BPs进行准确定性定量。

## Abstract (en)

Bisphenol A (BPA) is one of the most widely produced compounds in the world and was listed as a substance of very high concern by the European Chemicals Agency in 2016. Because of its toxicity, many countries and regions, including China, have banned BPA addition in feeding-bottles. And the European Union (EU) has banned BPA use in other food contact materials and thermal paper. Restrictions on BPA have contributed to the widespread use of alternatives. As the toxicity of BPA alternatives continues to be revealed, the alternatives of BPA alternatives are being developing. As the most extensive alternative for BPA, bisphenol S (BPS) was proven to have estrogen-disrupting effects and developmental toxicity of the neuroendocrine system. Therefore, BPS alternatives are used in thermal paper. In this study, alternatives to both BPA and BPS are collectively referred to as bisphenols (BPs). As a pooling matrix of many indoor chemicals, dust is an important pathway for human exposure to BPs. BPA and its alternatives are routinely detected in dust. As BPS alternatives have been detected in recycled paper and sludge, it is also very important to detected in dust. However, common analytical methods for BPs have low sensitivity and contain few BPS alternatives. Therefore, a high-throughput, high-accuracy, and high-sensitivity method must be established for the determination of BPs in dust; this will lay the foundation for subsequent studies on the environmental behavior and exposure risk of BPs.

In this study, an ultra performance liquid chromatography-tandem mass spectrometry (UPLC-MS/MS) method was developed for the simultaneous determination of 26 variations of BPs in dust. UPLC-MS/MS parameters of the variations were optimized to compare the separation effect and response intensity in different columns and mobile phases. The influence of the extraction solvent and solid phase extraction (SPE) on the extraction efficiency and purification effect of target compounds were optimized by using the isotopic internal standard method, and the 26 variations of BPs in dust was quantitatively analyzed. Finally, the dust samples were extracted by using 3 mL of acetonitrile and 3 mL of a 50% methanol aqueous solution in an ultrasound bath. The combined extract was further purified by using an Oasis HLB cartridge (60 mg/3 mL). The cartridge was then washed with a 40% methanol aqueous solution (0.5 mL) and eluted with methanol (2 mL). The target compounds were separated on a CORTECS^®^ UPLC^®^ C_18_ column (100 mm×2.1 mm, 1.6 μm), with methanol and 1 mmol/L ammonium fluoride solution as mobile phases and a flow rate of 0.3 mL/min. Electrospray ionization (ESI) was applied in the positive, negative, and multiple reaction monitoring (MRM) modes for the mass scan. Under optimized conditions, the linear ranges of the 26 targets behaved well linearly in their respective ranges with correlation coefficients (*r*^2^)>0.999. The limits of detection (LODs) and limits of quantification (LOQs) were assessed using signal-to-noise (*S/N*) ratios of 3 and 10, respectively. The LODs and LOQs of the method were 0.01-0.75 μg/kg and 0.02-2.50 μg/kg, respectively. The accuracy of the method was evaluated by conducting a recovery test at three spiked levels: LOQ, two times the LOQ, and 10 times the LOQ, with the average recoveries ranging from 83.7% to 114.9%. The precision of the method was evaluated by using the relative standard deviation (RSD). The intra-day RSDs and inter-day RSDs were 0.86%-9.79% (*n*=6) and 5.16%-19.5% (*n*=6), respectively. The established method was used to determine 11 dust samples. Fifteen BPs were detected at a detection rate of 9.1%-100.0%. The detection rate for BPA, BPS, bisphenol F (BPF), 4-hydroxy-4'-isopropoxydiphenylsulfone (BPSIP), and diphenyl sulfone (DPS) was 100.0%. BPSIP, 4-allyloxy-4'-hydroxydiphenyl sulfone (BPS-MAE), and bis-(3-allyl-4-hydroxyphenyl) sulfone (TGSA) were first detected in Chinese dust, whereas 4-benzyloxy-4'-hydroxydiphenyl sulfone (BPS-MPE), 4-hydroxybenzoic acid benzyl (PHBB), and DPS were first detected in dust samples worldwide. This method is simple, rapid, and sensitive, and is suitable for the qualitative screening and quantitative analysis of the 26 BPs in dust samples.

双酚A(BPA)是全球产量最大的化合物之一,由于其内分泌干扰效应^[1,2]^, 2016年被欧洲化学品管理局列为高度关注的物质^[3]^;包括我国在内的多个国家和地区禁止在婴幼儿奶瓶中添加BPA,欧盟随后陆续在其他食品接触材料和热敏纸中禁止使用BPA。尽管如此,BPA的需求量仍在增加,2020年全球产量已超过700万吨,并以4.3%的年增长率上升^[4]^。另一方面,BPA的限制也促进了其替代物的广泛使用,目前报道的替代物已超过50种。例如双酚S(BPS)、双酚F(BPF)和双酚AF(BPAF)等被用于聚碳酸酯塑料和环氧树脂的生产中^[5⇓-7]^;卤代衍生物四氯双酚A(TCBPA)和四溴双酚A(TBBPA)等被用于阻燃剂生产中^[8]^。这些替代物可以通过灰尘、饮用水以及食品等多种途径进入人体,且具有与BPA类似或更强的毒性^[9,10]^,如BPS、BPF等对性激素、甲状腺激素和神经内分泌激素也具有干扰效应,能影响机体生殖功能、性腺发育、神经行为并且导致激素依赖性疾病的发生和发展^[11⇓-13]^。随着BPA替代物毒性被不断揭示,BPA替代物的替代物也不断发展。作为BPA最广泛的替代物,BPS被证实具有雌激素干扰作用和神经内分泌系统发育毒性^[14]^,因此BPS替代物例如2,4'-二羟基二苯砜(2,4-BPS)、双(3-烯丙基-4-羟基苯基)砜(TGSA)、4-烯丙氧基-4'-羟基二苯砜(BPS-MAE)、4-苯氧苯基-4'-羟基苯基砜(BPS-MPE)和4-羟基-4'-异丙氧基二苯砜(BPSIP)等也被用于热敏纸生产中^[15]^。目前关于BPS替代物的毒性研究较少,有限的毒理学实验表明BPSIP具有类似BPA和BPS的雌激素干扰作用^[16,17]^。本文将BPA、BPA替代物和BPS替代物统称为双酚类化合物(BPs)。

近年来,环境介质中的BPs受到广泛关注,灰尘作为许多室内化学物质的汇集介质,是人类暴露BPs的一种重要途径^[11,12]^。目前灰尘中BPs的检测方法主要集中于BPA及BPA替代物,常用的分析方法有荧光分析法^[18]^、气相色谱-质谱法(GC-MS)^[19,20]^和液相色谱-串联质谱法(LC-MS/MS)^[21⇓-23]^。其中LC-MS/MS由于选择性和灵敏度高,已成为检测BPs最常用的方法。BPS替代物已在再生纸^[24]^和污泥^[25]^中被广泛检出,然而灰尘中这类物质的检测方法较少:Duenas-Mas等^[26]^使用1-己醇-四氢呋喃-水(1∶1∶4, v/v/v)提取结合LC-MS/MS的方法测定了灰尘中6种BPS替代物,检出限(LOD)为0.50~10.00 μg/kg,该方法覆盖靶标物质少,检测灵敏度低。

本研究选择BPA、BPA替代物(包括BPS、双酚E(BPE)、BPF、双酚T(BPT)、双酚Z(BPZ)、BPAF、双酚AP(BPAP)、双酚芴(BPFL)、双酚TMC(BPTMC)、4,4'-二羟基二苯醚(DHDPE)、一氯双酚A(MCBPA)、3,3-二氯双酚A(3,3-DCBPA)、3,5-二氯双酚A(3,5-DCBPA)、三氯双酚A(TriCBPA)、TCBPA和TBBPA)及BPS替代物(包括2,4-BPS、BPSIP、BPS-MAE、BPS-MPE、TGSA、4-羟基苯甲酸苯甲酯(PHBB)、双(2-氯乙基)醚-4,4'二羟基二苯基砜共聚物(D-90(*n*=1)、二苯砜(DPS)和双(4-烯丙氧基苯基)砜(BPS-DAE))等26种BPs为目标化合物,通过对色谱-质谱和前处理条件进行优化,建立了一种高通量、高准确度和高灵敏度的检测方法,为后续研究BPs的环境行为和暴露风险奠定基础。

## 1 实验部分

### 1.1 仪器与试剂

ACQUITY^TM^ UPLC超高效液相色谱仪、Oasis HLB固相萃取柱(SPE, 60 mg/3 mL)(美国Waters公司)、Sciex QTRAP^®^-6500三重四极杆质谱仪(美国SCIEX公司); Vortex-Genies 2涡旋振荡器(美国Scientific Industries公司); N-EVAPTM-116氮吹仪(美国Organomation公司); Milli-Q超纯水机(美国Millipore公司)。

BPA、BPF、BPS、2,4-BPS、BPAF、TCBPA、BPE和TBBPA(纯度均>98%)均购自日本Tokyo Chemical Industry公司;BPT、BPZ、BPFL、BPTMC、BPAP和DHDPE(纯度均>99%),氟化铵、甲醇和乙腈为色谱纯,均购自美国Sigma公司;D-90(*n*=1)、MCBPA、3,3-DCBPA、3,5-DCBPA、TriCBPA、TGSA和BPS-MAE(纯度均>98%)均购自加拿大Toronto Research Chemicals公司;BPSIP、DPS、PHBB、BPS-DAE和BPS-MPE(纯度均>98%)均购自德国Dr. Ehrenstorfer公司;BPA-^13^C_12_、BPF-^13^C_12_、BPS-^13^C_12_、BPAF-d_4_、TCBPA-^13^C_12_和TBBPA-^13^C_12_(纯度均>99%)购自美国剑桥同位素实验室;BPE-^13^C_6_、BPZ-^13^C_12_、BPAP-^13^C_6_、2,4-BPS-d_8_、BPSIP-d_7_和PHBB-^13^C_6_(纯度均>99%)购自德国Dr. Ehrenstorfer公司。

### 1.2 标准溶液的配制

对于固体标准品,分别精确称取10.0 mg(精确至0.1 mg),转移至10.0 mL棕色量瓶中,用甲醇溶解,室温下超声10 min,用甲醇定容至刻度,配制成质量浓度为1.0 mg/mL的标准储备液,4 ℃冰箱冷藏保存。

对于液体标准品,直接用甲醇稀释为1.0 mg/mL的标准储备液,4 ℃冰箱冷藏保存。使用时用甲醇逐级稀释,配制成质量浓度为0.1、1.0、10.0和100.0 μg/L的系列混合标准工作液。

内标标准品采用同样的方法配制,使用时用甲醇稀释成25.0 μg/L的混合内标工作液。

### 1.3 样品前处理

准确称取50.0 mg(±0.5 mg)灰尘样品,加入25.0 μg/L内标工作液10 μL,涡旋混匀,室温放置2 h,加入3 mL乙腈,超声10 min, 4 ℃、10000 r/min离心10 min。上清液转入15 mL离心管,残渣加入3 mL 50%甲醇水溶液进一步提取,合并两次提取液,加入6 mL超纯水,混合均匀后上样于Oasis HLB SPE柱(60 mg/3 mL,使用前依次用10 mL甲醇和5 mL水活化)。用0.5 mL 40%甲醇水溶液淋洗SPE柱,用2 mL甲醇洗脱,洗脱液氮气吹干后,用50 μL 50%甲醇水溶液复溶,待测。

### 1.4 仪器条件

#### 1.4.1 色谱条件

色谱柱:CORTECS^®^ UPLC^®^ C_18_色谱柱(100 mm×2.1 mm, 1.6 μm);流动相A: 1 mmol/L氟化铵水溶液,流动相B:甲醇;柱温:40 ℃;流速:0.3 mL/min;进样量:5 μL。梯度洗脱程序:0~0.5 min, 35%B; 0.5~1.5 min, 35%B~50%B; 1.5~5.0 min, 50%B~100%B; 5.0~7.0 min, 100%B; 7.0~7.1 min, 100%B~35%B; 7.1~9.1 min, 35%B。

#### 1.4.2 质谱条件

离子源:电喷雾电离(ESI)源;离子源温度:500 ℃;扫描方式:正离子和负离子模式同时切换扫描;喷雾电压:5500 V(ESI^+^)/-4500 V(ESI^-^);辅助气1:379225 Pa;辅助气2:379225 Pa;气帘气:241325 Pa;碰撞气:N_2_;碰撞气压力:Medium。其他参数包括保留时间、定量与定性离子对、碰撞能量(CE)及去簇电压(DP)见表1。

**表1:** 26种BPs及内标化合物的质谱参数

Compound		Retention time/min		Precursor ion (*m/z*)		Daughter ion (*m/z*)		Collision energy/eV		Declustering potential/V		IS
Bisphenol S (BPS)		1.90		249		108^*^		-35		-80		BPS-^13^C_12_
						92		-35		-80		
2,4'-Dihydroxydiphenyl sulfone (2,4-BPS)		2.26		249		107.9^*^		-34		-60		2,4-BPS-d_8_
						156		-22		-60		
4,4'-Dihydroxydiphenyl ether (DHDPE)		2.49		201		108^*^		-21		-60		BPF-^13^C_12_
						173		-21		-60		
Bisphenol F (BPF)		2.96		199		93^*^		-34		-80		BPF-^13^C_12_
						105		-30		-80		
Bisphenol E (BPE)		3.27		213		198^*^		-32		-80		BPE-^13^C_6_
						197		-22		-60		
Bisphenol T (BPT)		3.28		217		124^*^		-29		-50		BPS-^13^C_12_
						185		-30		-50		
Bis(2-chloroethyl)ether-4,4'-dihydroxydiphenyl		3.36		569		411^*^		-36		-80		BPS-^13^C_12_
sulfone copolymer (D-90(*n*=1))						249.1		-35		-100		
4-Allyloxy-4'-hydroxydiphenyl sulfone (BPS-MAE)		3.42		289		248^*^		-38		-60		BPS-^13^C_12_
						184		-20		-50		
Bisphenol A (BPA)		3.54		227		212^*^		-26		-60		BPA-^13^C_12_
						133		-29		-100		
4-Hydroxy-4'-isopropoxydiphenyl sulfone (BPSIP)		3.55		291		248^*^		-30		-80		BPSIP-d_7_
						184		-35		-100		
Bis-(3-allyl-4-hydroxyphenyl) sulfone (TGSA)		3.72		329		132^*^		-43		-120		BPS-^13^C_12_
						148		-37		-120		
Tetrachlorobisphenol A (TCBPA)		3.73		329		250^*^		-42		-120		TCBPA-^13^C_12_
						278		-33		-120		
Monochloro bisphenol A (MCBPA)		3.90		261		182^*^		-42		-100		BPA-^13^C_12_
						167		-50		-100		
4-Hydroxybenzoic acid benzyl (PHBB)		3.93		227		92^*^		-27		-60		PHBB-^13^C_6_
						136		-23		-60		
4-Benzyloxy-4'-hydroxydiphenyl sulphone		4.00		339		248^*^		-27		-60		BPS-^13^C_12_
(BPS-MPE)						108		-42		-100		
Bisphenol AF (BPAF)		4.01		335		265^*^		-25		-60		BPAF-d_4_
						197		-56		-60		
Bisphenol AP (BPAP)		4.07		289		274^*^		-29		-100		BPAP-^13^C_6_
						195		-40		-50		
3,3-Dichloro bisphenol A (3,3-DCBPA)		4.20		297		246^*^		-36		-60		BPA-^13^C_12_
						218		-23		-100		
3,5-Dichloro bisphenol A (3,5-DCBPA)		4.21		297		216^*^		-41		-100		BPA-^13^C_12_
						244		-34		-100		
Bisphenol FL (BPFL)		4.24		349		256^*^		-36		-100		BPA-^13^C_12_
						215		-36		-120		
Bisphenol Z (BPZ)		4.27		267		173^*^		-37		-100		BPZ-^13^C_12_
						223		-37		-120		
3,3',5-Trichloro bisphenol (TriCBPA)		4.48		331		252^*^		-43		-120		BPA-^13^C_12_
						280		-36		-80		
Bisphenol TMC (BPTMC)		4.86		309		215^*^		-42		-120		BPA-^13^C_12_
						200		-34		-100		
Tetrabromobisphenol A (TBBPA)		4.93		545		420^*^		-50		-120		TBBPA-^13^C_12_
						448		-54		-120		
diphenyl sulfone (DPS)		3.09		219		77^*^		30		100		BPS-^13^C_12_
						141		30		80		
Bis(4-allyloxyphenyl)sulfone (BPS-DAE)		4.33		331		197^*^		24		120		BPS-^13^C_12_
						79		37		120		
BPS-^13^C_12_		1.90		261		114		-35		-100		
2,4-BPS-d_8_		2.26		257		112		-31		-60		
BPF-^13^C_12_		2.96		211		99		-30		-80		
BPE-^13^C_6_		3.27		219		204		-27		-80		
BPA-^13^C_12_		3.54		239		224		-25		-80		
BPSIP-d_7_		3.55		298		250		-26		-100		
TCBPA-^13^C_12_		3.73		661		440		-45		-120		
PHBB-^13^C_6_		3.93		233		98		-25		-60		
BPAF-d_4_		4.01		339		269		-24		-60		
BPAP-^13^C_6_		4.07		295		280		-29		-100		
BPZ-^13^C_12_		4.27		279		235		-37		-80		
TBBPA-^13^C_12_		4.93		838		617		-50		-120		

* Quantitative ion.

### 1.5 质量控制

实验前,先对15 mL离心管、超纯水与有机溶剂等进行背景考察,均未检测到目标化合物。为避免前处理过程污染,每次实验同时做3个程序空白,与样品一起处理,26种目标化合物的含量在程序空白样品中均低于LOD。

## 2 结果与讨论

### 2.1 色谱-质谱条件优化

首先对26种BPs的质谱条件进行优化,包括扫描方式、CE和DP等参数,使每种化合物的母离子与特征碎片离子强度达到最大,将响应强度最大的碎片离子设为定量离子,次级响应离子设为定性离子。其中DPS与BPS-DAE两种物质仅在正离子模式下有响应,其余24种化合物在负离子模式下响应较高,因此选择正负离子同时扫描的方式进行采集。

由于目标化合物具有疏水性(log *K*_ow_ 1.65~6.08),本研究比较了3种反相色谱柱BEH C_18_(100 mm×2.1 mm, 1.7 μm)、HSS C_18_(100 mm×2.1 mm, 1.8 μm)和CORTECS C_18_(100 mm×2.1 mm, 1.6 μm)。BEH C_18_色谱柱是通用型反相色谱柱;HSS C_18_色谱柱是硅胶基体C_18_柱,比BEH C_18_柱保留能力更强;CORTECS C_18_色谱柱是基于固体核心颗粒的反相色谱柱,对中性、酸性或复杂体系的样品提供较好分离。结果发现,26种BPs在3种色谱柱上均能分离且获得较好的峰形,但使用HSS C_18_色谱柱分离时目标化合物响应较低;BPA及BPA替代物(除BPS)使用BEH C_18_色谱柱和CORTECS C_18_色谱柱分离均可获得较高的响应且差别不大,但是BPS及其替代物在CORTECS C_18_色谱柱分离时的信噪比(*S/N*)比BEH C_18_色谱柱高1.1~3.9倍,因此本研究选择CORTECS C_18_色谱柱进行分离分析。

实验在此基础上进一步比较了26种BPs在不同流动相组成条件下的响应强度。首先以甲醇作为有机相,比较了水相为超纯水、0.1%甲酸水溶液、0.1%乙酸水溶液和0.5 mmol/L氟化铵水溶液时的响应值。水相为超纯水时26种BPs的响应值都很低;对于BPA及其替代物(除BPS),水相为0.1%甲酸水溶液、0.1%乙酸水溶液和0.5 mmol/L氟化铵水溶液时响应值较超纯水提高1.1~21.1倍,且差别不大;对于BPS及其替代物,水相为0.5 mmol/L氟化铵水溶液时的响应值比另外两种溶液高1.3~5.5倍。进一步比较了氟化铵水溶液浓度为0.5、1和2 mmol/L时的响应值,结果显示,氟化铵水溶液浓度为1 mmol/L时几乎所有物质的响应值都最高,因此选择1 mmol/L氟化铵水溶液为水相。在此基础上,又比较了有机相为甲醇或乙腈时的响应值,有机相为甲醇时26种BPs的响应值比乙腈高1~4.7倍,因此选择甲醇-1 mmol/L氟化铵水溶液作为流动相。空白灰尘样品中加入26种BPs标准品(5.0 μg/L)的总离子流色谱图见图1。

**图1:**
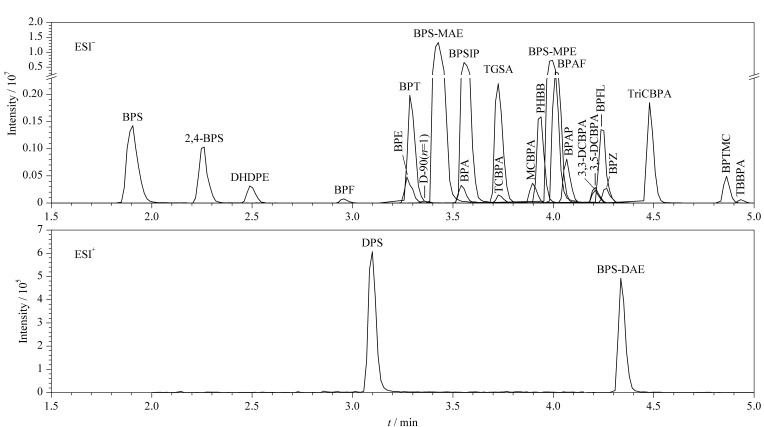
空白加标样品中26种BPs(5.0 μg/L)的总离子流色谱图

### 2.2 前处理条件的优化

#### 2.2.1 提取溶剂的优化

26种BPs的极性范围跨度较大,因此本文分别用3 mL的乙腈、甲醇、50%甲醇水溶液和80%甲醇水溶液对目标物进行提取。结果显示,26种BPs用这4种溶剂提取的回收率分别为14.6%~86.5%、16.1%~76.2%、23.1%~75.4%和14.2%~79.0%。其中TBBPA、TCBPA、TriCBPA、3,3-DCBPA、3,5-DCBPA和TGSA这6种化合物用乙腈提取的回收率(14.6%~58.9%)低于50%甲醇水溶液(61.5%~78.4%)和80%甲醇水溶液(57.6%~71.1%),其他20种化合物用乙腈提取的回收率均最高(50.6%~86.5%)。因此本文进一步比较了用3 mL乙腈提取后再用3 mL乙腈、50%甲醇水溶液或80%甲醇水溶液二次提取的回收率。结果如图2所示,在26种化合物中有17种化合物使用3 mL 50%甲醇水溶液二次提取的回收率最高,另外9种化合物中只有BPFL的回收率(61.4%)偏低,使用80%甲醇水溶液二次提取的回收率(63.8%)并未明显提高,其余8种化合物的回收率为76.0%~103.5%,可以满足实验需求。综上所述,本研究选择3 mL乙腈加3 mL 50%甲醇水溶液作为提取溶剂。

**图2:**
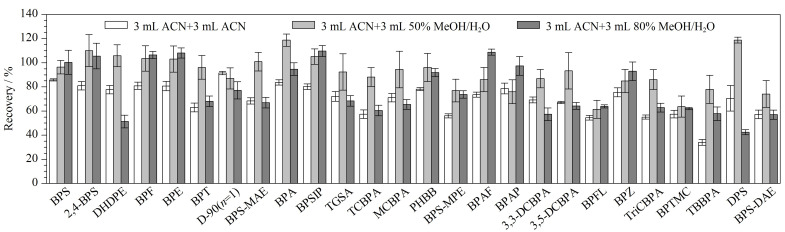
采用不同提取液时26种BPs的回收率(*n*=3)

#### 2.2.2 固相萃取条件的优化

本研究参考Yang等^[23]^的方法,选择Oasis HLB SPE柱对上样液进行净化。一些化合物由于极性较强,提取液直接上样时26种BPs在SPE柱上的回收率仅为39.0%~83.3%。为了增加目标化合物在SPE柱上的保留,用6 mL超纯水对提取液进行稀释,此时26种BPs的回收率为65.4%~99.8%,回收率得到明显改善。用12 mL超纯水进行稀释后回收率为67.8%~97.1%,增加超纯水体积,回收率没有进一步改善。因此选用6 mL超纯水稀释上样,此时上样液体积为12 mL。

对上样后的SPE柱进行淋洗能够减少干扰物对目标化合物的影响,因此在不影响回收率的情况下选择合适的淋洗液至关重要。未淋洗时,BPF和D-90(*n*=1)的峰形较差,有明显杂峰,使用0.5 mL 40%甲醇水溶液淋洗后杂峰减少,响应增加,此时26种BPs的回收率为68.6%~99.1%,与未淋洗时无明显区别。将淋洗液体积增至1 mL, BPF和D-90(*n*=1)的信号无明显增强,而BPS、2,4-BPS、BPS-DAE和BPTMC这4种化合物的回收率低于40%。因此,本研究选择0.5 mL 40%甲醇水溶液作为淋洗液。

### 2.3 基质效应

基质效应是指共流出干扰物对目标离子造成基质抑制或增强的效应,基质效应在质谱分析中普遍存在,会影响分析结果的准确性^[27]^。本文中,基质效应的计算公式为ME=(空白灰尘基质经前处理后加入标准物质得到的峰面积/50%甲醇水溶液中直接加入标准物质得到的峰面积-1) ×100%^[28,29]^。|ME|≤20%,表明无明显基质效应;20%<|ME|≤50%,表明基质效应中等;|ME|>50%,表明对目标化合物有较强的基质效应。实验结果表明,26种BPs的基质效应为0.5%~54.6%,有13种化合物无明显基质效应,10种化合物基质效应中等,3种化合物基质效应较强。为消除基质效应的影响,本实验采用同位素内标法进行定量。

### 2.4 方法学考察

#### 2.4.1 线性范围和检出限

对于有同位素内标的化合物采用对应的同位素内标进行定量,对于无同位素内标的化合物,采用化学结构、保留时间、回收率和基质效应均相近的同位素内标进行定量,详见表1。配制质量浓度分别为0.02、0.05、0.25、0.5、1.00、2.50、5.00、10.00、25.00和50.00 μg/L,内标质量浓度为5.00 μg/L的系列混合标准溶液。经UPLC-MS/MS测定,以26种BPs的定量离子峰面积与内标峰面积之比为纵坐标,质量浓度为横坐标,绘制工作曲线,内标法定量。结果如表2所示,26种待测物在各自的范围内线性关系良好,相关系数(*r*^2^)均大于0.999。在空白灰尘样品中添加标准品,以定量离子*S/N*为3或10的质量浓度分别确定方法的LOD和LOQ,方法的LOD为0.01~0.75 μg/kg, LOQ为0.02~2.50 μg/kg。与文献[23,26,30,31]测定灰尘中BPs的方法相比,本方法的灵敏度显著提高。

**表2:** 26种BPs的线性范围、线性方程、相关系数、检出限、定量限、回收率和精密度(*n*=6)

Compound	Linear range/(μg/L)	Linear equation	*r*^2^	LOD/ (μg/kg)	LOQ/ (μg/kg)	Recoveries/%			Intra-day RSD/%	Inter-day RSD/%
						LOQ	2LOQ	10LOQ		
BPS	0.10-50	*Y*=0.09684*X*+0.08569	0.9999	0.03	0.10	109.6	92.3	100.9	2.12	5.41
2,4-BPS	0.25-50	*Y*=0.04661*X*+0.00363	0.9994	0.08	0.25	98.9	101.3	100.2	2.54	6.93
DHDPE	0.50-50	*Y*=0.01898*X*+0.00304	0.9992	0.15	0.50	98.5	101.4	100.1	5.45	8.24
BPF	2.50-50	*Y*=0.09588*X*+0.00842	0.9999	0.75	2.50	100.6	101.2	100.6	3.42	16.5
BPE	0.50-50	*Y*=0.06569*X*+0.01917	0.9999	0.15	0.50	101.9	100.1	102.5	4.17	6.68
BPT	0.10-50	*Y*=0.81667*X*+0.02810	0.9995	0.03	0.10	97.5	100.2	83.7	5.59	11.6
D-90(*n*=1)	2.50-50	*Y*=0.00212*X*+3.10855	0.9999	0.75	2.50	99.7	97.2	96.4	5.80	6.37
BPS-MAE	0.02-50	*Y*=0.76816*X*+0.02221	0.9995	0.01	0.02	105.1	97.8	101.4	1.44	7.13
BPA	0.50-50	*Y*=0.13014*X*+0.05599	0.9992	0.15	0.50	99.9	100.5	122.2	1.78	19.5
BPSIP	0.02-50	*Y*=0.71425*X*+0.06079	0.9995	0.02	0.05	103.5	99.8	100.4	3.67	5.79
TGSA	0.05-50	*Y*=1.03445*X*+0.12088	0.9998	0.02	0.05	101.2	102.9	100.3	2.16	11.5
TCBPA	0.05-50	*Y*=1.03451*X*+0.14530	0.9995	0.02	0.05	93.1	100.3	96.3	3.20	7.09
MCBPA	0.20-50	*Y*=0.14550*X*+0.01068	0.9991	0.06	0.20	99.2	99.9	100.4	2.33	7.35
PHBB	0.10-50	*Y*=0.08620*X*+0.01208	0.9994	0.03	0.10	100.1	100.3	97.9	3.58	7.12
BPS-MPE	0.05-50	*Y*=0.46659*X*+0.02538	0.9998	0.02	0.05	83.5	108.5	100.1	1.74	7.28
BPAF	0.05-50	*Y*=0.12337*X*+0.01175	0.9992	0.02	0.05	99.5	101.2	99.1	4.24	7.60
BPAP	0.10-50	*Y*=0.04528*X*+0.00553	0.9991	0.03	0.10	99.2	99.4	95.3	1.76	9.77
3,3-DCBPA	0.20-50	*Y*=0.08545*X*+0.01279	0.9997	0.06	0.20	98.3	100.1	100.5	1.32	9.87
3,5-DCBPA	0.20-50	*Y*=0.12149*X*+0.01597	0.9999	0.06	0.20	99.7	100.2	101.5	1.09	7.88
BPFL	0.10-50	*Y*=0.53395*X*+0.03229	0.9994	0.03	0.10	102.1	100.2	103.3	0.86	5.31
BPZ	0.20-50	*Y*=0.12559*X*+0.01768	0.9996	0.06	0.20	99.9	100.2	99.2	2.27	6.87
TriCBPA	0.05-50	*Y*=0.68358*X*+0.09214	0.9994	0.02	0.05	89.9	99.1	100.2	1.77	6.22
BPTMC	0.20-50	*Y*=0.17473*X*+0.02196	0.9996	0.06	0.20	100.3	100.6	98.6	1.16	5.16
TBBPA	1.00-50	*Y*=0.02251*X*+0.00201	0.9992	0.33	1.00	100.5	99.1	114.9	9.79	16.3
DPS	0.20-50	*Y*=0.03380*X*+0.00671	0.9997	0.06	0.20	101.1	99.2	87.2	2.10	17.0
BPS-DAE	0.50-50	*Y*=0.02505*X*+0.00209	0.9995	0.15	0.50	99.7	102.2	99.2	2.29	7.72

*Y*: ratio of the peak area of compounds to peak area of internal standard; *X*: mass concentration, μg/L.

#### 2.4.2 准确度和精密度

方法的准确度采用回收率评价,选择空白灰尘样本分别在LOQ、2倍LOQ和10倍LOQ 3个水平进行加标回收试验,每组6个平行,方法的精密度以日内相对标准偏差(RSD)和日间RSD评价。选择10倍LOQ加标水平进行5天日间RSD测定,回收率和RSD如表2所示。方法的回收率为83.7%~114.9%,日内RSD为0.86%~9.79%,日间RSD为5.16%~19.5%。表明本方法具有良好的准确性和精密度。

### 2.5 实际样品测定

采用本研究建立的方法,对周边生活区11个灰尘样本进行检测(见表3)。对于检测浓度超过标准曲线线性范围的样品,重新称量,按倍数增加内标浓度,根据1.3节步骤处理后用相同倍数的50%甲醇水稀释后再上机检测。结果表明,26个BPs中检出15种化合物,检出率为9.1%~100.0%,其中BPA、BPF、BPS、BPSIP和DPS等5种化合物检出率为100.0%, BPSIP、BPS-MAE和TGSA 3种化合物首次在国内灰尘中检出,BPS-MPE、PHBB和DPS这3种化合物在灰尘中首次检出,结果提示灰尘中BPS替代物亟须引起关注。

**表3:** 11份灰尘样品中BPs的含量

Compound	DF/%	Median/ (μg/kg)	Minimum/ (μg/kg)	Maximum/ (μg/kg)	Compound	DF/%	Median/ (μg/kg)	Minimum/ (μg/kg)	Maximum/ (μg/kg)	
BPS	100.0	11.57	0.92	206.66	2,4-BPS	72.7	0.45	<LOD	6.15	
DHDPE	90.9	0.56	<LOD	1.48	BPF	100.0	82.35	32.78	287.88	
BPT	9.1	0.13	<LOD	0.13	TBBPA	81.8	2.83	<LOD	27.10	
BPS-MAE	45.5	0.83	<LOD	1.98	BPA	100.0	365.92	56.79	1851.80	
BPSIP	100.0	0.32	0.08	27.47	TGSA	27.3	1.17	<LOD	2.18	
MCBPA	63.6	1.09	<LOD	1.97	PHBB	90.9	0.73	<LOD	3.76	
BPS-MPE	63.6	0.20	<LOD	67.75	BPAF	54.6	1.28	<LOD	2.82	
DPS	100.0	4.50	2.25	77.22						

DF: detection frequency.

## 3 结论

本研究通过对色谱-质谱参数、色谱柱、流动相、提取溶剂和固相萃取条件优化,建立了超高效液相色谱-串联质谱法检测灰尘中26种BPs残留量的方法。应用所建立的方法,对实际样品进行测定,初步发现BPS替代物在灰尘样品中广泛存在。与其他方法相比,本方法目标物种类多,尤其覆盖了新兴BPS替代物,灵敏度高,适用于灰尘样品中26种BPs的定性筛查和定量分析,为研究其在灰尘中的残留和风险评估提供了可靠的技术参考。
